# ICTV Virus Taxonomy Profile: Phenuiviridae 2023

**DOI:** 10.1099/jgv.0.001893

**Published:** 2023-09-13

**Authors:** Takahide Sasaya (笹谷孝英), Gustavo Palacios, Thomas Briese, Francesco Di Serio, Martin H. Groschup, Yutaro Neriya (煉谷裕太朗), Jin-Won Song (송진원), Yasuhiro Tomitaka (冨髙保弘)

**Affiliations:** 1 Institute for Plant Protection, National Agriculture and Food Research Organization, Tsukuba, Japan; 2 Icahn School of Medicine at Mount Sinai, New York, USA; 3 Columbia University, New York, USA; 4 Institute for Sustainable Plant Protection, National Research Council, Bari, Italy; 5 Institute of Novel and Emerging Infectious Diseases, Friedrich-Loeffler-Institut, Greifswald-Insel Riems, Germany; 6 School of Agriculture, Utsunomiya University, Utsunomiya, Japan; 7 Korea University, Seoul, Republic of Korea

**Keywords:** *Bunyavirales*, ICTV Report, *Phenuiviridae*, phlebovirus, taxonomy, tenuivirus

## Abstract

The family *Phenuiviridae* comprises viruses with 2–8 segments of negative-sense or ambisense RNA, comprising 8.1–25.1 kb in total. Virions are typically enveloped with spherical or pleomorphic morphology but can also be non-enveloped filaments. Phenuivirids infect animals including livestock and humans, birds, plants or fungi, as well as arthropods that serve as single hosts or act as biological vectors for transmission to animals or plants. Phenuivirids include important pathogens of humans, livestock, seafood and agricultural crops. This is a summary of the International Committee on Taxonomy of Viruses (ICTV) Report on the family *Phenuiviridae*, which is available at ictv.global/report/phenuiviridae.

## Virion

**Fig. 1.**
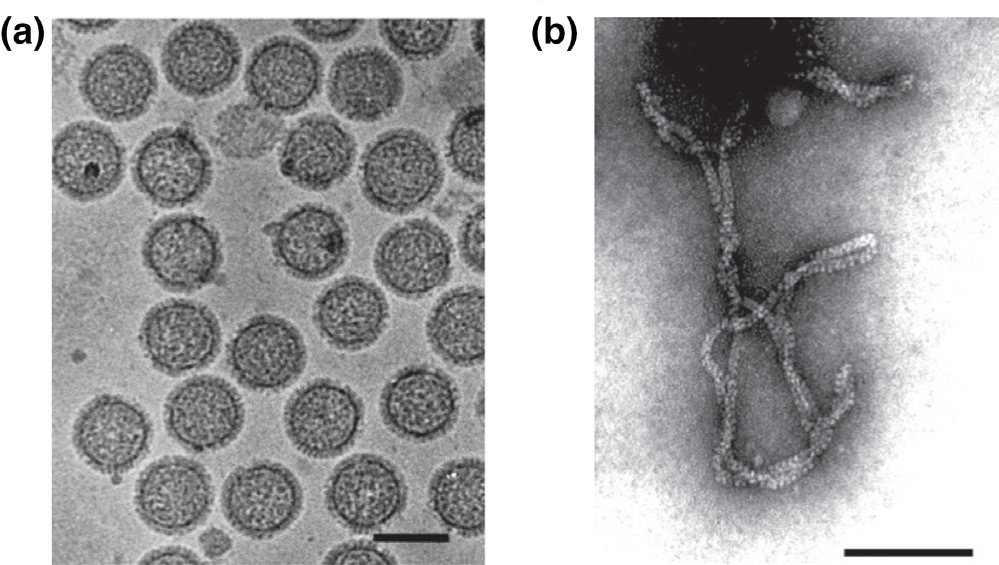
(**a**) Cryo-electron micrograph of purified uukuvirus particles (Uukuniemi virus). Bar, 100 nm (courtesy of C.-H. von Bornsdorff). (**b**) Electron micrograph of purified tenuivirus particles (rice hoja blanca virus). Bar, 100 nm (courtesy of A. M. Espinoza).

Members of the family with enveloped virions mainly infect vertebrates and invertebrates; virions are usually spherical or pleomorphic, 8–120 nm in diameter with glycoprotein surface projections (Table 1, Fig. 1). Members of the family without envelopes mainly infect plants and fungi as well as vector arthropods; virions are spiral-shaped filaments 2.0–2.5 nm in diameter.

**Table 1.** Characteristics of members of the family *Phenuiviridae*

Example	Rift Valley fever virus (S, DQ380151; M, DQ380206; L, DQ375403), species *Phlebovirus riftense*, genus *Phlebovirus*
Virion	Enveloped spherical or pleomorphic virions 80–120 nm in diameter, or non-enveloped filaments
Genome	Two to eight negative-sense or ambisense RNA molecules ranging from 0.8 to 9.8 kb
Replication	Ribonucleoprotein (RNP) complexes containing full-length anti-genomic RNAs serve as templates for synthesis of nascent RNP complexes containing genomic RNAs
Translation	From capped mRNAs that lack poly(A) termini. The 5′-cap structure is derived from cellular mRNAs via cap-snatching
Host range	Vertebrates including mammals and birds, invertebrates, plants and fungi
Taxonomy	Realm *Riboviria*, phylum *Negarnaviricota*, class *Ellioviricetes*, order *Bunyavirales*; >20 genera including >150 species

## Genome

**Fig. 2.**
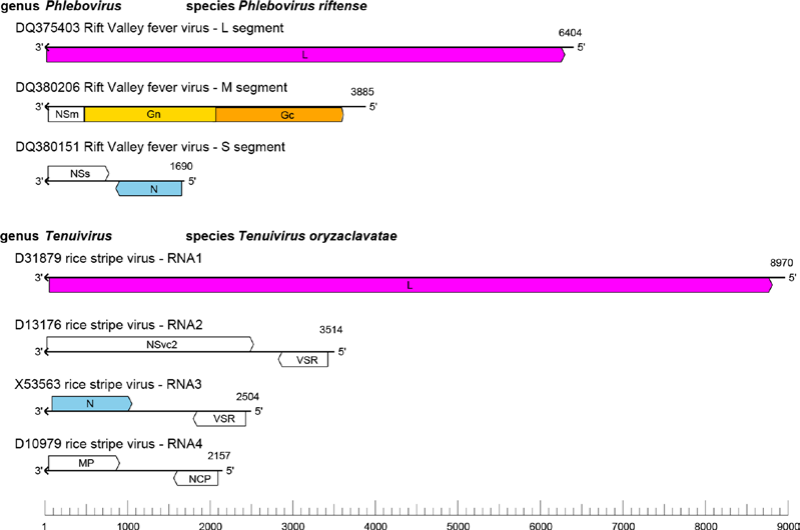
Genome organization of phenuivirids with (*Phlebovirus*) and without (*Tenuivirus*) enveloped virions. L, large protein; Gn, Gc, glycoproteins; NS, non-structural protein; N, nucleocapsid protein; VSR, viral suppressor of RNA silencing; MP, movement protein; NCP, major non-capsid protein.

The phenuivirid genome (Fig. 2) consists of 2–8 negative-sense or ambisense RNA molecules, comprising a total of 8.1–25.1 kb. The segments are termed the large (L) segment or RNA1, the medium (M) segment or RNA2, the small (S) segment or RNA3, and additional fourth or more RNAs. These RNAs encode a structural nucleocapsid protein (N), one or two glycoproteins (Gn, Gc; not encoded by all members) and large protein (L) in the virus-complementary sense. Some members also encode up to ten non-structural proteins (NS).

## Replication

Virions attach to the host cell through unknown cell-surface receptors and enter through clathrin-mediated endocytosis. Fusion of the viral Gc protein fusion peptide with endosomal membranes facilitates the release of ribonucleoprotein (RNP) complexes into the cytoplasm. The 3′- and 5′-ends of each RNA serve as promoters for both mRNA and antigenome synthesis. Viral mRNAs are truncated relative to the viral RNA, they are not polyadenylated and possess 5′-methylated caps derived from host cellular mRNAs. Proteins are translated on free ribosomes or membrane-bound ribosomes. The Gn and Gc proteins are generated by co-translational cleavage and targeted to and retained in the Golgi complex. RNP complexes are targeted near the Golgi complex. Genomes are packaged by signals from non-conserved sequences in the terminal untranslated regions. Virions bud into Golgi cisternae and are transported to the cell surface by the secretory pathway. In the case of phenuivirids that lack glycoproteins, the mechanisms of virion attachment, host cell entrance, genome packaging and virion budding are unknown [1,3].

## Taxonomy

Current taxonomy: ictv.global/taxonomy. Viruses assigned to each genus form a monophyletic clade based on phylogenetic analyses of L protein sequences. Members of the genera *Bandavirus* and *Phlebovirus* are transmitted by ticks and sandflies, and infect mammals including humans in which they can cause fatal thrombocytopenia fever and other diseases. Members of the genus *Uukuvirus* are transmitted by ticks and infect mammals and birds. A member of the genus *Tanzavirus* has been detected by high-throughput sequencing of RNA from a human with fever. Members of the genera *Citricivirus*, *Goukovirus* and *Phasiviru*s infect invertebrates. The viruses assigned to the genera *Beidivirus*, *Horwuvirus*, *Hudivirus*, *Hudovirus*, *Ixovirus*, *Mobuvirus* and *Pidchovirus* have been detected in RNA from invertebrates. Members of the genera *Coguvirus*, *Mechlorovirus*, *Rubodvirus* and *Tenuivirus* are transmitted by either arthropods or grafting, and infect plants associated with diseases of agricultural importance. Members of the genera *Entoviru*s, *Laulavirus* and *Lentinuvirus* have been found in fungi [4,6].

## Resources

Full ICTV Report on the family *Phenuiviridae*: ictv.global/report/phenuiviridae.

## Abbreviations

G: glycoprotein
L: large protein
N: nucleocapsid
NS: non-structural protein
RNP: ribonucleoprotein
